# Wearable Technologies in Head and Neck Oncology: Scoping Review

**DOI:** 10.2196/72372

**Published:** 2025-10-10

**Authors:** Matthew Shammas-Toma, Gianluca Sampieri, Michael Xie, Aishwaria Maxwell, Alex Esemezie, Quynh Pham, Joseph A Cafazzo, Philip Wong, C Jillian Tsai, David P Goldstein, John R de Almeida, Ervin Sejdic, Christopher M K L Yao

**Affiliations:** 1 Temerty Faculty of Medicine, University of Toronto, Toronto, ON, Canada; 2 Department of Otolaryngology - Head and Neck Surgery, University of Toronto, Toronto, ON, Canada; 3 Department of Otolaryngology - Head and Neck Surgery, Princess Margaret Cancer Center, University Health Network, 610 University Avenue, Toronto, ON, M5G 2M9, Canada, 1 4163403063; 4 Toronto General Hospital, Center for Digital Therapeutics, University Health Network, Toronto, ON, Canada; 5 Department of Radiation Oncology, Princess Margaret Cancer Center, University Health Network, Toronto, ON, Canada; 6 Edward S. Rogers Department of Electrical and Computer Engineering, Faculty of Applied Science and Engineering, University of Toronto, Toronto, ON, Canada; 7 North York General Hospital, Toronto, ON, Canada

**Keywords:** wearable technology, head and neck cancer, digital health, therapeutics, vocal rehabilitation, otorhinolaryngologic diseases

## Abstract

**Background:**

Head and neck cancer (HNC) survivors face profound functional and quality-of-life deficits due to disease- and treatment-related sequelae, ranging from mild fatigue to debilitating dysphagia. Wearable technology, by monitoring biometric data such as step counts or providing swallowing biofeedback, offers a unique method for tracking and monitoring the negative effects of HNC.

**Objective:**

The aim of this study was to explore the current applications of wearable technology in HNC.

**Methods:**

A scoping review was conducted following the PRISMA-ScR (Preferred Reporting Items for Systematic Reviews and Meta-Analyses Extension for Scoping Reviews) guidelines. A search strategy was built, and a literature search was performed across 5 databases. The initial search yielded 5256 studies, which underwent a 2-phase screening process: title and abstract review followed by full-text review. Inclusion criteria included peer-reviewed, English-language articles published between January 2002 and April 2024 that used wearable technology in HNC care. After full-text review, 9 studies met the inclusion criteria. Data were manually extracted and synthesized narratively.

**Results:**

The included studies examined 3 main types of wearable devices: radioactivity (2 studies), physical activity (4 studies), and throat physiology monitors (3 studies). Radioactivity monitors detected residual radioactivity and thyroidal radioiodine uptake. They demonstrated potential to reduce radioactivity exposure risk and personalize radiation doses for patients with thyroid cancer. Physical activity monitors tracked step counts, heart rate, and sleep habits. Low step counts were significantly associated with increased anxiety, radiation-related toxicity, hospital admission rates, and feeding tube placement. One study also linked poor sleep patterns to declines in quality of life. Throat physiology monitors measured pharyngeal electromyography data as well as extrinsic laryngeal muscle movements. Throat sensors achieved high accuracy in classifying swallowing events and translating muscle movements into speech. While earliest in the development continuum, they are promising tools for swallowing and vocal rehabilitation therapy. Barriers to wearable adoption included wearable discomfort, technical difficulties, and patient withdrawal due to treatment side effects. As the definition of wearable adherence varied widely, we propose that future studies report wearable adherence as “percentage of prescribed wear time achieved” to facilitate cross-study comparisons.

**Conclusions:**

Wearable technology may enhance treatment monitoring, prognostication, and rehabilitation in head and neck oncology. Radioactivity and physical activity monitors provide actionable insights for clinical decision-making, while throat physiology monitors offer innovative solutions for speech and swallowing therapy. However, challenges such as device adherence, data integration, and patient comfort must be addressed to realize their full potential. Future research should prioritize larger, longitudinal studies, standardized adherence metrics, and consider the integration of artificial intelligence to refine predictive capabilities. By overcoming these barriers, wearable technology could transform survivorship care, improving functional outcomes and quality of life for patients with HNC.

## Introduction

Head and neck cancer (HNC) had the eighth highest incidence of any cancer in the world, accounting for over 700,000 new cases globally in 2022 [1]. HNCs arise in the upper aerodigestive tract and can affect patients’ ability to interact with the world, including breathing, swallowing, and speaking. Furthermore, treatment toxicities can lead to significant changes to patients’ physical and psychosocial quality of life (QOL) [2]. While visits with clinicians may detect recurrence, they are unable to assess the full spectrum of needs for patients with HNC [3,4].

In recent years, wearable technology has emerged as a promising tool to address some of these challenges. These are portable devices equipped with sensor technologies to enable real-time tracking of health data including but not limited to: vital sign monitoring, daily step counts, sleep quality, and more. Wearable technologies have been used for safety monitoring, early and real-time diagnosis, chronic disease optimization, and patient engagement [5]. Within oncology, wearable technology has demonstrated benefit in prognostication, treatment monitoring, and rehabilitation. Examples in the literature include improving physical activity among patients with breast cancer [6], and predicting readmission after peritoneal cancer surgery [7].

Wearable data can improve performance status assessment to guide treatment selection and flag patients at heightened risk of treatment-related toxicities [8-10]. These insights are particularly valuable for HNC survivors, who often experience functional decline driven by fatigue, xerostomia, hoarseness, dysphagia, and shoulder weakness [11,12]. For example, by quantifying early dysphagia events, wearable sensors may prompt timely swallowing therapy or dietary interventions. Similarly, step counters can flag declining physical activity in patients struggling with fatigue, and shoulder-mounted sensors may support physiotherapy in those with accessory nerve weakness. These applications highlight the head and neck as a practical site for wearable technology. Herein, our aim is to explore the current applications of wearable technology in head and neck oncology.

## Methods

This scoping review was conducted according to PRISMA-ScR (Preferred Reporting Items for Systematic Reviews and Meta-Analyses Extension for Scoping Reviews) 2020 guidelines [13]. A health science researcher (AM) conducted a literature search on PubMed, Embase, OVID Medline, CINAHL Plus, and the Cochrane Database of Systematic Reviews for studies published between January 2002 and April 2024, focusing on recent advances in head and neck oncology [14]. A combination of controlled vocabularies and free-text keywords was used (Table S1-S6 in Multimedia Appendix 1). Two reviewers (MS and AM) conducted a snowballing search to find additional citations.

Inclusion criteria consisted of peer-reviewed, English-language journal articles and full-length conference papers that used wearable technology applicable to HNC care. More specifically, we sought to review the use of wearable technology in all aspects of HNC care (ie, prevention, diagnosis, treatment, and surveillance). Wearable studies involving other cancer types were included if they presented data specific to patients with HNC. Reviews and meta-analyses were excluded, but their reference lists were manually searched for key articles.

All title, abstract, and full-text screening was completed using Covidence (Covidence systematic review software; Veritas Health Innovation). Search titles and abstracts were independently screened by 2 reviewers (MS and AM). Full articles were retrieved and independently reviewed. Disagreements were resolved by consensus and in consultation with a third reviewer (CY).

A narrative synthesis was conducted to synthesize the different wearable technologies reported in the HNC literature. All data were charted manually. Data were charted using the following categories: authors, study year, geographic location, journal, study design, HNC type, sample size, participant mean age, participant sex, follow-up duration, HNC treatment, timing of wearable use relative to HNC treatment, wearable type, wearable parameters, study objective, study outcomes, wearable clinical impact, and explicit barriers to wearable use. The barriers to use were based on the listed reasons for participant withdrawal and poor adherence. Data were extracted by 2 reviewers (MS and GS) and independently verified with a third reviewer (CY).

## Results

### Study Selection

**Figure 1.**
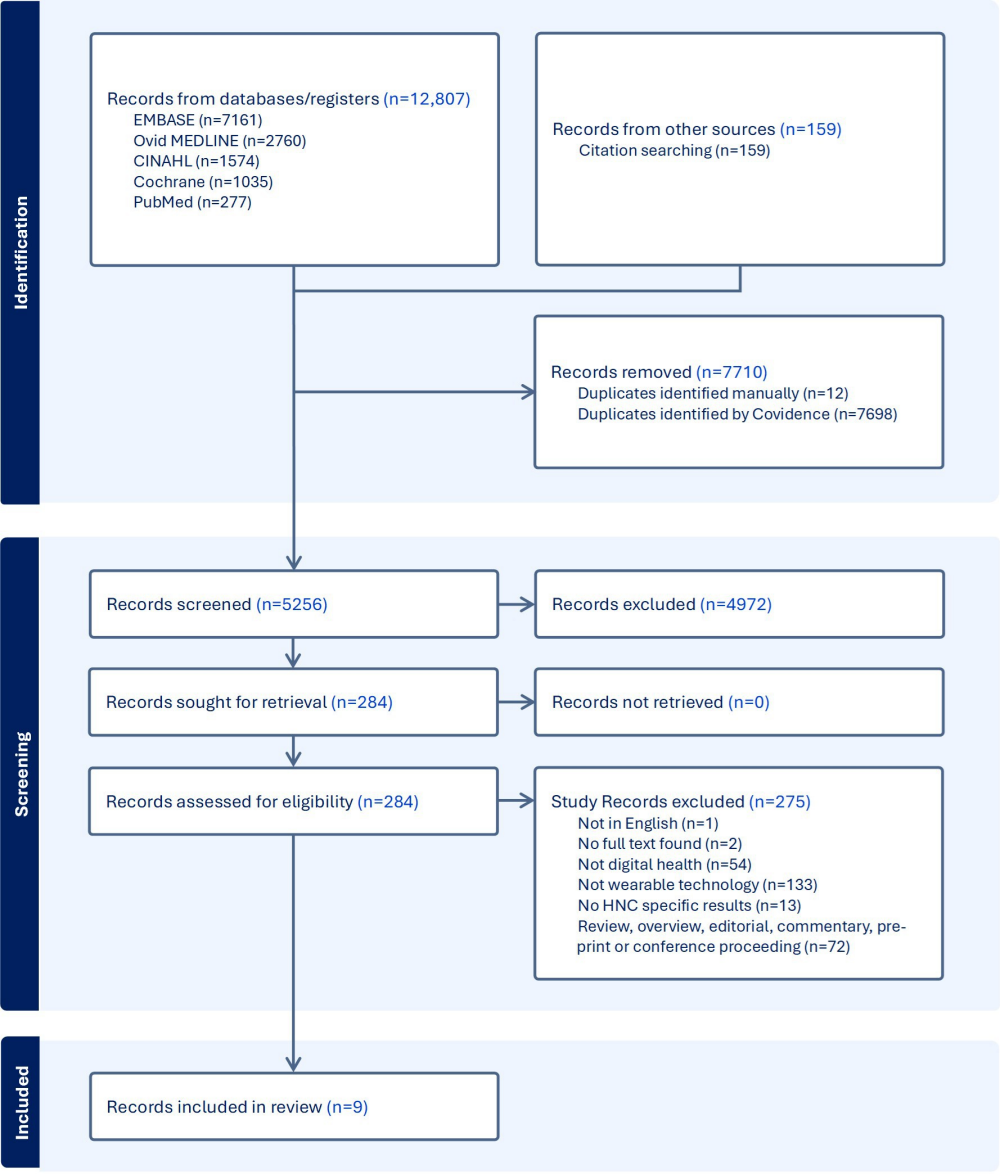
Preferred Reporting Items for Systematic Reviews and Meta-Analyses flowchart depicting study identification and screening. Preferred Reporting Items for Systematic Reviews and Meta-Analyses flowchart illustrating the study selection process detailing databases used as well as studies identified, screened, assessed for eligibility, and included in the final review.

Initial database search yielded 5256 studies (Figure 1). After title and abstract screening, 284 studies met criteria for full-text review. The reduction from an initial pool of 5256 studies to 284 studies is primarily attributable to a broad search strategy which included all forms of digital health across head and neck oncology from 2002 onward (Table S1-S6 in Multimedia Appendix 1). However, the vast majority of studies did not include wearable devices and wearables were not studied in HNC prior to 2018. After a full-text review, 271 studies were excluded. We excluded studies that did not end up using wearable technology (n=185), were reviews (n=72), were not applicable to HNC care (n=13), had no full text available (n=2), and were not reported in the English language (n=1). After exclusion, a total of 9 studies met the inclusion criteria for this review [15-23].

### Study Characteristics

Table 1 illustrates the characteristics of the 9 studies published from 2018 to 2024 [15-23]. Of the 9 articles included in this review, there were 3 observational prospective cohort studies [18-20], 3 feasibility studies [16,17,21], 2 validation studies [22,23], and 1 case-control study [15]. Five studies [18-22] examined wearables for various HNC types while 4 studies [15-17,23] examined wearables for specific HNC types. The specific HNC types included well-differentiated thyroid carcinoma, as well as oral cavity, oropharyngeal, and laryngeal cancer.

**Table 1.** Wearable study characteristics.

Source	Study design	HNC^a^ type	Sample size (n)	Mean age (range)	Sex, n (%)	Follow-up duration	Treatment	Treatment phase
Constantinescu 2018, Canada, Dysphagia [15]	Case-control study	Oral cancer Oropharyngeal cancer	20	57.85 (41-65)	Male: 14 (70) Female: 6 (30)	N/A^b^	Surgery, RT,^c^ or CRT^d^	During and post
Gallicchio 2020, Italy, Journal of Translational Engineering in Health and Medicine [16]	Feasibility study	Differentiated thyroid carcinoma	20	41 (33‐49)	Male: 8 (40) Female: 12 (60)	2.3 days	Radionuclide therapy	During
Santhanam 2021, USA, Nature Scientific Reports [17]	Feasibility study	Papillary and Hurthle cell thyroid carcinoma	2	46 (37‐55)	Male: 2 (100) Female: 0 (0)	NA	Radionuclide therapy	During and post
Boeke 2022, Germany, Nature Scientific Reports [18]	Observational prospective cohort	Various HNCs	19	69 (68-75)	Male: 12 (63) Female: 7 (37)	46 days	RT or CRT	During
Sher 2022, USA, Technical Innovations and Patient Support in Radiation Oncology [19]	Observational prospective cohort	Various HNCs	43	61 (54‐69)	Male: 37 (86) Female: 6 (14)	72 days	RT or CRT	During
Ohri 2023, USA, JCO Clinical Care Informatics [20]	Observational prospective cohort	Various HNCs	29	61 (45‐78)	Male: 24 (83) Female: 5 (17)	40 days	RT or CRT	During
Holländer-Mieritz 2023, Denmark, JCO Clinical Care Informatics [21]	Feasibility study	Various HNCs	10	62 (50‐75)	Male: 8 (80) Female: 2 (20)	60 days	RT or CRT	During
Xu 2023, China, Cancers [22]	Validation study	Various HNCs	N/A	N/A	N/A	N/A	N/A	N/A
Che 2024, USA, Nature Communications [23]	Validation study	Laryngeal cancer	N/A	N/A	N/A	N/A	N/A	N/A

a HNC: head and neck cancer.

b Not applicable.

c RT: radiotherapy.

d CRT: chemoradiotherapy.

The median sample size was 20 with a range of 2‐43. The overall mean participant age among the studies was 57 (SD 8.58) years. Studies predominantly included male participants with a mean of 74% (SD 17.84). Patients were followed with wearables for a median of 21 days (IQR 5-40). Seven studies [15-21] investigated wearables in a clinical setting while 2 studies [22,23] focused on the development of new wearables. Of the 7 studies [15-21], 5 investigated wearables during patient treatment [16-20] and 2 investigated wearables during treatment and posttreatment [15,21] (Table 1).

### Types of Wearable Devices Used in HNC Care

Table 2 summarizes the different types of wearable devices used in HNC care across the 9 studies [15-23]. There were 3 throat physiology monitor studies [15,22,23], 2 radioactivity monitor studies [16,17], and 4 physical activity monitor studies [18-21]. Two radioactivity monitor studies followed patients during radionuclide therapy [16,17] (Table 1). One study aimed to detect residual radioactivity [16], whereas the second study aimed to detect thyroidal radioiodine uptake (Table 2) [17].

All 4 physical activity monitor studies were implemented around patients’ radiotherapy or chemoradiotherapy treatment courses [18-21] (Table 1). There have been no studies around the time of surgery. These studies monitored step counts, with 2 tracking heart rate and 1 tracking sleeping habits (Table 2) [18-21].

Three studies assessed externally applied throat physiology monitors. Xu et al [22] developed a general throat sensor that incorporates a 3-axis accelerometer and surface electromyography data. Che et al [23] developed a throat sensor that measures extrinsic laryngeal muscle activity to predict intended speech through machine learning. Finally, Constantinescu et al [15] studied the performance of a throat physiology monitor that detects patient swallowing using submental surface electromyography data to track home-based swallowing therapy (Table 2).

**Table 2.** Wearable technology parameters, outcomes, and response rate*.*

Source	Wearable technology	Parameters measured	Purpose	Outcomes	Clinical impact	Barriers to use
Constantinescu 2018 [15]	Throat physiology monitor	Submental sEMG^a^	Remote detection of swallowing for home-based swallowing therapy	92.7% sensitive for swallowing detection in patients with HNC^b^; Swallowing duration, frequency, and power were significantly lower for patients with HNC	Automated feedback during home-based swallowing therapy	Surgical reconstruction of submental area; History of stroke
Gallicchio 2020 [16]	Radioactivity monitor	Residual radioactivity	Detect residual radioactivity	Remote monitor correlates with home monitor (*r*=0.96)^c^	Operational efficiency and reduced exposure risk for medical staff	Not specified
Santhanam 2021 [17]	Radioactivity monitor	Thyroid radioiodine uptake	Detect thyroidal radioiodine uptake	Remote monitor correlates with gold-standard gamma probes (*r*=0.87)^c^	Potential to increase the accuracy of radioiodine therapy dosing	Not specified
Boeke 2022 [18]	Physical activity monitor	Step count	Relationship between daily step counts with QOL^d^ and treatment-related toxicities	Step count decline during RT^e^ and cetuximab treatment^c^; Step counts increase post-RT^c^; Step count decline correlated with QOL decline	Low step count may predict QOL and treatment-related toxicities	Not specified
Sher 2022 [19]	Physical activity monitor	Step count, heart rate, sleep habits	Relationship between step counts, heart rate, and sleep with QOL, hospital admissions, and change in pain medication	Step count decline precedes hospital admission and QOL decline by 1 week^c^; Less sleep precedes QOL decline by 1 week^c^	Step count and sleep predict hospital admission and QOL decline by 1 week	Severe treatment-related side effects Wearable discomfort
Ohri 2023 [20]	Physical activity monitor	Step count	Relationship between daily step counts with hospitalization and PROs^f^	Low step counts were associated with increased anxiety and sadness^c^; High average daily step counts were associated with a reduced risk of hospitalization and reduced risk of feeding tube placement^c^	Low step count predicts patient mood and risk of hospitalization	Severe treatment-related side effects Wearable discomfort Permanent data loss
Holländer-Mieritz 2023 [21]	Physical activity monitor	Step count, heart rate	Describe the variations in step count and heart rate during a RT/CRT^g^ course	One patient had declining heart rate correlated with decreased step count; The second patient had stable heart rates and step counts	Conclusions were limited by poor adherence	Patient technical difficulties Demanding to charge the smartwatch
Xu 2023 [22]	Throat physiology monitor	3-axis accelerometer sEMG	Remote diagnosis and treatment evaluation	92% accuracy in predicting coughing, drinking, swallowing, and talking states	Swallowing therapy and postsurgical rehabilitation	NA
Che 2024 [23]	Throat physiology monitor	Laryngeal muscle movements	Speech for patients with dysfunctional or absent vocal cords	94.68% accuracy in translating laryngeal muscle movements into voice signals; No change in wearable temperature or sound pressure level after 40 minutes use	Voice restoration for patients with dysfunctional or absent vocal cords	NA

a sEMG: surface electromyography.

b HNC: head and neck cancer.

c statistical significance.

d QOL: quality of life.

e RT: radiotherapy.

f PRO: patient-reported outcome.

g CRT: chemoradiotherapy.

### Effectiveness of Wearable Devices in HNC Care

Wearable radioactivity monitors correlated well with conventional measurements of residual radioactivity and thyroid radioactivity reuptake [16,17]. In the study by Gallicchio et al [16], a wearable radioactivity monitor measured residual radioactivity following radioactive iodine treatment in patients with thyroid cancer. Compared to a conventional installed room device, their wearable radioactivity monitor was found to significantly reduce staff radioactivity exposure levels and promote safe discharge home for patients. In the study by Santhanam et al [17], a collarbone radioactivity monitor provided more detailed information on thyroid radioiodine uptake compared to standard gamma probes, including higher sampling frequency, fractional uptake distribution over time, and differences in uptake lateralization.

Physical activity monitors provided evidence for tracking step counts. Across 3 studies, low step counts were associated with several clinical outcomes [18-20]. In the study by Boeke et al [18], step counts decreased with increasing radiotherapy toxicity in all but 1 patient. Sher et al [19] identified that step count decline was significantly associated with a reduction in QOL and hospital admission 1 week later in 25 patients actively undergoing radiotherapy. Moreover, this was the only study in which the wearable device monitored sleep patterns using movement and heart rate tracking, whereby fewer total minutes of recorded sleep were associated with lower QOL scores. In a third study by Ohri et al [ 20], high daily step count was significantly associated with a reduced risk of feeding tube placement and hospitalization, while low step counts were significantly associated with anxiety and depression.

Xu et al [22] and Che et al [23] both developed stretchable external throat sensors. The wearable by Xu et al [22] could measure respiratory rate and heart rate as well as predict the patient’s state (vocalization, coughing, drinking, swallowing, or talking) using a machine learning algorithm with a limited set of classified outcomes. The external throat sensor investigated by Che et al [23] offers a novel method for laryngeal speech generation by detecting extralaryngeal muscle activity and using a machine learning algorithm to predict intended speech. Collectively, while originally developed to classify throat movement and predict speech, these throat sensor platforms hold significant clinical promise. Potential applications include supporting swallowing therapy, evaluating aspiration risk, assessing rehabilitation adherence, quantifying aphonia burden, and facilitating voice restoration.

Constantinescu et al [15] used a throat sensor to predict patient swallowing events for remote swallowing therapy using a classification algorithm. The device achieved an acceptable sensitivity and positive predictive value for swallowing detection (Table 2). Moreover, a portable swallowing detector can provide patients with live biofeedback and physicians with real-time updates, acting as a powerful adjunctive tool for monitoring at-home swallowing therapy [15].

### Barriers to Use of Wearable Devices in HNC Care

Among the 9 studies [15-23], 4 directly assessed wearable feasibility and adherence [18-21]. Boeke et al [18] identified a higher rate of adherence at 92%, whereas the remaining 3 studies did not reach predetermined feasibility targets (80%‐90%) for adherence [19,20], or found a low rate of adherence (31%) [21]. However, the definition of adherence varied.

Notably, Sher et al [19] was the only study to examine wearable adherence during sleep. They found that patients wore wearables 60.5% of all potential sleep time, and only 1 patient was 100% adherent during sleep.

The common themes for participant study withdrawal were wearable discomfort, patient technical difficulties, and noncompliance due to treatment-related side effects (Table 2). Patient technical difficulties and reasons for wearable discomfort were not elaborated further in any study. With regard to treatment-related side effects, Sher et al [19] found that malnutrition and failure to thrive moderately correlated with participant study withdrawal.

## Discussion

### Principal Findings

Wearable technologies have emerged as an effective tool for health care delivery. In oncology, these technologies have been geared toward the phases of prognostication, treatment monitoring, and rehabilitation planning [24]. HNC treatment can have a profound impact on a patient’s physical function, receptive and expressive communication, swallowing, and overall QOL. As such, there are many existing and novel applications of wearable technology in this patient population. Herein, we identified 9 studies that explored 3 areas of wearable technology within HNC: radioactivity, physical activity, and throat physiology monitors [15-23]. Their clinical applications include improving radionuclide therapy delivery, prognostication of clinical outcomes, as well as speech and swallowing rehabilitation.

In this review, wearable devices allowed for accurate assessment of residual radioactivity and fractional uptake over time and localization after radioactive iodine treatment [16,17]. Step counts and physical activity monitoring were found to be associated with chemoradiotherapy-related toxicity, reduced QOL, mood, increased rates of hospital admission, and feeding tube placement [18-20]. Similarly, wearable device studies in other disease sites have also identified step counts to be associated with frailty, increased hospital readmission rates, treatment-related toxicity, prolonged length of stay in hospital, and death [25-28]. Regarding wearable adherence, participant adherence ranged from 31% to 92% [18-21]. However, these studies differ in their definitions of adherence and compliance between studies. This highlights the need for establishing standard definitions for wear time, as suggested by Beauchamp et al [9] and Huang et al [29]. We recommend that future wearable studies define and report adherence as “percentage of prescribed wear time achieved,” as shown in the equation below.

Throat physiology monitors, while earlier in the development continuum than other wearable monitors, represent an example where novel sensor technologies allow for unique wearable technology applications in HNC. For one, muscle activity sensors applied externally to the neck can measure extrinsic laryngeal muscle activity, which is then processed by a pretrained machine learning algorithm to predict anticipated words, enabling laryngeal speech [23]. While the study itself had a limited set of trained outputs, it establishes the groundwork for a novel voice rehabilitation approach. Similarly, Constantinescu et al [15] developed a wireless external throat sensor that rests on the submental area and provides patients with biofeedback during swallowing therapy exercises. While these technologies offer significant promise, further studies demonstrating clinical utility and feasibility are needed. In particular, throat sensor wearable adherence has not been studied. This represents an opportunity for future study, especially by assessing whether device type influences wearable adherence. Ongoing development of these technologies that focus on scalability and cost-minimization would further the potential benefit to patients with HNC worldwide.


$$Adherence\;\%=\;\frac{actual\;wear\;time\;\left(h\right)}{prescribed\;wear\;time\;\left(h\right)}\;x\;100\%$$

### Barriers

Barriers to integrating wearable technology in patients with HNC can be framed as technical factors, patient-related factors, and provider-related factors. Technical factors include the resources needed to troubleshoot devices, short battery life, and poor data management [20,21]. Patient-related factors include an initial device learning curve, wearable discomfort, and withdrawal from use due to severe chemotherapy- or radiotherapy-related side effects [19,20]. While provider-related factors were not explicitly discussed in the studies we identified, it is known that implementation from health care practitioners and stakeholders is often key to introducing these novel technologies [30].

An important component of provider-driven implementation is the integration of wearable data with existing electronic medical records. A 2024 wearable feasibility study involving 20 cancer patients demonstrated that Apple Watch data from 95% of participants was successfully transmitted to an electronic medical record and reviewed by an oncologist [31]. By automating data transfer, the undue burden to physicians and patients was reduced. This proof of concept lays the groundwork for scalable, low-burden integration of wearable data into oncology practice.

### Data Security and Privacy

Management of wearable device health data storage, device to database information transfer, and privacy varied between studies when reported. Like other personal health information, health data from wearable devices should be held to the same standards of confidentiality. Five studies used commercial devices [16,18-21]. Of these, 2 used in-house software and direct data transfer [16,18], 2 used commercial application programming interfaces with personal or study-provided smartphones for data storage and subsequent data syncing [19,20], and 1 study used an independent industry application programming interface with secure cloud servers for storage and data transfer [21]. Remaining studies used in-house developed devices and software.

While the technical methods of data storage and transfer vary widely, a consistent ethical standard must be applied. Two foundational principles emerge: informed consent and the right to withdraw. Patients should be fully informed about the nature of data collection, how and where their information is stored, and with whom it may be shared. Furthermore, patients must retain the ability to withdraw their data without penalty. These ethical obligations are particularly pressing in studies using third-party commercial devices, where data ownership and access are often unclear.

A cross-sectional survey of commercial wearable device users identified that over 50% of patients understood that wearable device health information is confidential in nature; however, their understanding of personal data handling and the expected privacy standards was lacking [32]. In this review, many of these wearables used third-party commercial devices and software. As wearable devices are implemented within research and clinical settings, the importance of educating patients on wearable device health data privacy and ensuring secure data storage and transfer must be clarified.

### Future Directions

A notable gap in the HNC wearable literature is the investigation of physical activity monitors during surgery. By contrast, physical activity monitoring in colorectal cancer has been studied in the preoperative, perioperative, and postoperative phases [33]. Surgery represents the main treatment for many patients with HNC, with surgery often being lengthy, complicated, and requiring a prolonged period of recovery. In turn, this represents a unique opportunity whereby physical activity monitors may aid in surgical risk assessment, acute postoperative monitoring, and beyond [34].

Another critical limitation is the small sample sizes across the included studies, which ranged from 2 to 43 participants. As a result, the current findings should be interpreted as hypothesis-generating rather than sufficient to inform clinical decision-making. Large randomized controlled trials are needed to validate early findings and establish the clinical utility of wearables in HNC care.

A third gap in the current literature is the lack of longitudinal studies. Most existing studies were limited to the acute radiotherapy phase, with follow-up periods under 3 months (Table 1). Longitudinal studies could help establish predictive thresholds for late-onset dysphagia and hospital readmission while also tracking rehabilitation over time. These studies can help incorporate wearables into long-term survivorship care.

The integration of artificial intelligence (AI) with wearable technology data has significant promise [35]. Recent studies demonstrate that machine learning algorithms can leverage wearable technology to train risk stratification models [36], monitor tolerance and response to chemotherapeutics [37], detect biomarkers [38], as well as adhere to treatment regimens [39]. In this review, 2 studies integrated AI with external laryngeal sensor data to classify speech and swallowing states to facilitate laryngeal rehabilitation [22,23]. AI is likely to further enhance the capabilities offered by wearable technologies.

### Study Limitations

Our scoping review identified 9 studies which reflected various applications including physical activity monitors, radioactivity monitoring, and novel throat sensors [15-23]. While their inherent differences limit our ability for further analyses, this limitation reflects the current state of research in wearable technology for HNC and highlights the need for more studies in this field. Second, our review only included studies published in English, which could have led to the exclusion of relevant studies published in other languages.

### Conclusion

This scoping review provides the first comprehensive overview of wearable technology applications in HNC. The analysis of 9 studies reveals a diverse range of wearable applications and identifies potential future directions [15-23]. These technologies demonstrate potential benefit for risk stratification, treatment monitoring, and augmenting rehabilitation for patients with HNC. With continued research in wearable technologies, care must also be taken to define measures of adherence, understand clinical integration barriers, and establish standards for patient health data and privacy with wearable devices in research and clinical settings.

## Supplementary material

10.2196/72372 Multimedia Appendix 1 Search strategy.

10.2196/72372 Checklist 1 PRISMA-ScR checklist.

## Abbreviations

AI: artificial intelligence
HNC: head and neck cancer
PRIMSA-ScR: Preferred Reporting Items for Systematic Reviews and Meta-Analyses Extension for Scoping Reviews
QOL: quality of life
