# Supplementary material for: Wearable Technologies in Head and Neck Oncology: Scoping Review
Source: JMIR Mhealth Uhealth. 2025 Oct 10;13:e72372. doi: 10.2196/72372 (PMC12513687; doi:10.2196/72372)
Supplement: Multimedia Appendix 1 [file mhealth-v13-e72372-s001.docx]

## Appendix

**Table S1.** Ovid MEDLINE Daily and Ovid MEDLINE search strategy and results on April 7, 2024.

| # | Searches | Results |
| --- | --- | --- |
| 1 | exp "Squamous Cell Carcinoma of Head and Neck"/ or exp "Head and Neck Neoplasms"/ | 358643 |
| 2 | (head adj3 neck adj3 (cancer* or neoplasm*)).mp. | 84320 |
| 3 | (("upper aerodigestive tract" or uadt) adj4 (cancer* or neoplasm*)).mp. | 822 |
| 4 | ((digital or virtual) adj2 health adj3 intervention*).mp. | 1661 |
| 5 | exp Telemedicine/ or exp Mobile Applications/ or exp Internet/ or exp Wearable Electronic Devices/ | 170522 |
| 6 | (telehealth or Telecare or telemedicine or telediagnosis or digital or eHealth).mp. | 261294 |
| 7 | (app, mobile or app, portable electronic or app, portable software or app, smartphone or application, mobile or application, portable electronic or application, portable software or applications, mobile or apps, mobile or apps, smartphone or electronic app, portable or electronic application, portable or mobile app or mobile application or mobile applications or mobile apps or portable electronic app or portable electronic application or portable electronic applications or portable electronic apps or portable software app or portable software application or portable software applications or portable software apps or smartphone app or smartphone apps or software app, portable or software application, portable).mp. | 21248 |
| 8 | 1 or 2 or 3 | 370475 |
| 9 | 4 or 5 or 6 or 7 | 387199 |
| 10 | 8 and 9 | 1671 |
| 11 | limit 10 to yr="2002 -2024” | 1403 |

**Table S2.** Ovid MEDLINE: Epub Ahead of Print, In-Process & Other Non-Indexed Citations search strategy and results on April 7, 2024.

| # | Searches | Results |
| --- | --- | --- |
| 1 | exp "Squamous Cell Carcinoma of Head and Neck"/ or exp "Head and Neck Neoplasms"/ | 433735 |
| 2 | (head adj3 neck adj3 (cancer* or neoplasm*)).mp. | 88182 |
| 3 | (("upper aerodigestive tract" or uadt) adj4 (cancer* or neoplasm*)).mp. | 971 |
| 4 | ((digital or virtual) adj2 health adj3 intervention*).mp. | 1546 |
| 5 | exp Telemedicine/ or exp Mobile Applications/ or exp Internet/ or exp Wearable Electronic Devices/ | 235227 |
| 6 | (telehealth or Telecare or telemedicine or telediagnosis or digital or eHealth).mp. | 381347 |
| 7 | (app, mobile or app, portable electronic or app, portable software or app, smartphone or application, mobile or application, portable electronic or application, portable software or applications, mobile or apps, mobile or apps, smartphone or electronic app, portable or electronic application, portable or mobile app or mobile application or mobile applications or mobile apps or portable electronic app or portable electronic application or portable electronic applications or portable electronic apps or portable software app or portable software application or portable software applications or portable software apps or smartphone app or smartphone apps or software app, portable or software application, portable).mp. | 29644 |
| 8 | 1 or 2 or 3 | 442007 |
| 9 | 4 or 5 or 6 or 7 | 550057 |
| 10 | 8 and 9 | 3958 |
| 11 | limit 10 to yr="2002 -2024” | 3663 |

**Table S3.** CINAHL Database | EBSCO search strategy and results on April 7, 2024.

| # | Searches | Results |
| --- | --- | --- |
| 1 | (TI ( telehealth or Telecare or telemedicine or telediagnosis or Application or eHealth ) ) OR ( AB ( telehealth or Telecare or telemedicine or telediagnosis or Application or eHealth ) ) OR ( MW ( telehealth or Telecare or telemedicine or telediagnosis or Application or eHealth ) ) | 211,253 |
| 2 | AB ( head N3 neck N3 ( carcinoma* or cancer* or neoplasm*) ) OR TI ( head N3 neck N3 ( carcinoma* or cancer* or neoplasm*) ) OR MH ( head N3 neck N3 ( carcinoma* or cancer* or neoplasm*) ) | 16,915 |
| 3 | TI ( ("upper aerodigestive tract" or uadt) N3 (carcinoma* cancer* or neoplasm*) ) OR AB ( ("upper aerodigestive tract" or uadt) N3 (carcinoma* cancer* or neoplasm*) ) OR MH ( ("upper aerodigestive tract" or uadt) N3 (carcinoma* cancer* or neoplasm*) ) | 11 |
| 4 | TI ( (digital or virtual) health N3 intervention* ) OR AB ( (digital or virtual) health N3 intervention* ) OR MH ( (digital or virtual) health N3 intervention* ) | 389 |
| 5 | (MH "Assistive Technology Devices") OR (MH "Wearable Sensors") OR (MH "Mobile Applications") | 23,329 |
| 6 | S1 OR S4 OR S5 | 228,005 |
| 7 | S2 OR S3 | 16,921 |
| 8 | S6 AND S7 | 571 |

**Table S4.** *Cochrane* Library search strategy and results on April 7, 2024.

| # | Searches | Results |
| --- | --- | --- |
| 1 | ("head and neck squamous cell cancer"):ti,ab,kw | 133 |
| 2 | [mh "Head and Neck Neoplasms"] | 9434 |
| 3 | head NEAR/3 neck NEAR/3 (cancer* OR neoplasm*) | 7668 |
| 4 | ((digital OR virtual) NEAR/2 health NEAR/3 intervention*):ti,ab,kw | 424 |
| 5 | [mh "Telemedicine"] OR [mh "Mobile Applications"] OR [mh "Internet"] OR [mh "Electronic Wearable Devices"] | 12092 |
| 6 | (telehealth or Telecare or telemedicine or telediagnosis or Application or eHealth or mHealth or mobile NEXT app*):ti,ab,kw | 89525 |
| 7 | #1 OR #2 OR #3 | 13506 |
| 8 | #4 OR #5 OR #6 | 95671 |
| 9 | #7 AND #8 with Cochrane Library publication date Between Jan 2002 and Apr 2024 | 547 |

**Table S5.** Embase search strategy and results on April 7, 2024.

| # | Searches | Results |
| --- | --- | --- |
| 1 | exp "Squamous Cell Carcinoma of Head and Neck"/ or exp "Head and Neck Neoplasms"/ | 433735 |
| 2 | (head adj3 neck adj3 (cancer* or neoplasm*)).mp. | 88182 |
| 3 | (("upper aerodigestive tract" or uadt) adj4 (cancer* or neoplasm*)).mp. | 971 |
| 4 | ((digital or virtual) adj2 health adj3 intervention*).mp. | 1546 |
| 5 | exp Telemedicine/ or exp Mobile Applications/ or exp Internet/ or exp Wearable Electronic Devices/ | 235227 |
| 6 | (telehealth or Telecare or telemedicine or telediagnosis or digital or eHealth).mp. | 381347 |
| 7 | (app, mobile or app, portable electronic or app, portable software or app, smartphone or application, mobile or application, portable electronic or application, portable software or applications, mobile or apps, mobile or apps, smartphone or electronic app, portable or electronic application, portable or mobile app or mobile application or mobile applications or mobile apps or portable electronic app or portable electronic application or portable electronic applications or portable electronic apps or portable software app or portable software application or portable software applications or portable software apps or smartphone app or smartphone apps or software app, portable or software application, portable).mp. | 29644 |
| 8 | 1 or 2 or 3 | 442007 |
| 9 | 4 or 5 or 6 or 7 | 550057 |
| 10 | 8 and 9 | 3958 |
| 11 | limit 10 to yr="2002 -2024” | 3663 |

**Table S6.** PubMed search strategy and results on April 7, 2024.

| Searches | Results |
| --- | --- |
| ((((head and neck cancer*[Title/Abstract]) OR (head and neck neoplasm*[Title/Abstract]) OR (head and neck cancer[MeSH]) OR (head and neck neoplasm[MeSH]))) AND (((telemedicine[Title/Abstract]) OR (telemedicine[MeSH]) OR (telemedicine[Other Term]) OR (telehealth[Title/Abstract]) OR (telehealth[MeSH]) OR (telehealth[Other Term]) OR (telecare[Title/Abstract]) OR (telediagnosis[Title/Abstract]) OR ("virtual health intervention*"[Title/Abstract]) OR ("digital health intervention*"[Title/Abstract]) OR (eHealth[Title/Abstract]) OR (mHealth[Title/Abstract]) OR ("mobile application*"[Title/Abstract]) OR ("mobile app"[Title/Abstract]) OR ("electronic wearable device*"[Title/Abstract])))) Filters: from 2002 - 2024 | 277 |
